# Inhibition of StearoylCoA Desaturase Activity Blocks Cell Cycle Progression and Induces Programmed Cell Death in Lung Cancer Cells

**DOI:** 10.1371/journal.pone.0011394

**Published:** 2010-06-30

**Authors:** Daniel Hess, Jeffrey W. Chisholm, R. Ariel Igal

**Affiliations:** 1 Department of Nutritional Sciences and Rutgers Center for Lipid Research, Rutgers, the State University of New Jersey, New Brunswick, New Jersey, United States of America; 2 Biology, Gilead Sciences Inc., Palo Alto, California, United States of America; University of Barcelona, Spain

## Abstract

Lung cancer is the most frequent form of cancer. The survival rate for patients with metastatic lung cancer is ∼5%, hence alternative therapeutic strategies to treat this disease are critically needed. Recent studies suggest that lipid biosynthetic pathways, particularly fatty acid synthesis and desaturation, are promising molecular targets for cancer therapy. We have previously reported that inhibition of stearoylCoA desaturase-1 (SCD1), the enzyme that produces monounsaturated fatty acids (MUFA), impairs lung cancer cell proliferation, survival and invasiveness, and dramatically reduces tumor formation in mice. In this report, we show that inhibition of SCD activity in human lung cancer cells with the small molecule SCD inhibitor CVT-11127 reduced lipid synthesis and impaired proliferation by blocking the progression of cell cycle through the G_1_/S boundary and by triggering programmed cell death. These alterations resulting from SCD blockade were fully reversed by either oleic (18:1n-9), palmitoleic acid (16:1n-7) or cis-vaccenic acid (18:1n-7) demonstrating that cis-MUFA are key molecules for cancer cell proliferation. Additionally, co-treatment of cells with CVT-11127 and CP-640186, a specific acetylCoA carboxylase (ACC) inhibitor, did not potentiate the growth inhibitory effect of these compounds, suggesting that inhibition of ACC or SCD1 affects a similar target critical for cell proliferation, likely MUFA, the common fatty acid product in the pathway. This hypothesis was further reinforced by the observation that exogenous oleic acid reverses the anti-growth effect of SCD and ACC inhibitors. Finally, exogenous oleic acid restored the globally decreased levels of cell lipids in cells undergoing a blockade of SCD activity, indicating that active lipid synthesis is required for the fatty acid-mediated restoration of proliferation in SCD1-inhibited cells. Altogether, these observations suggest that SCD1 controls cell cycle progression and apoptosis and, consequently, the overall rate of proliferation in cancer cells through MUFA-mediated activation of lipid synthesis.

## Introduction

Non-small cell lung cancer is the leading cause of death by cancer in the developed world. The 5-year survival rate is ∼15% for patients with lung cancer, and decreases to ∼5% in subjects with metastatic cancer [1], therefore novel therapeutic approaches based on new molecular targets are needed. In recent years, studies have revealed that the constitutive activation of lipid biosynthesis, particularly the synthesis of saturated (SFA) and monounsaturated fatty acids (MUFA), is a critical event in carcinogenesis [2], [3], suggesting that lipogenic pathways may be valuable targets for cancer intervention. SCD is a family of Δ9-fatty acid desaturase isoforms that converts SFA into MUFA [4]. Two isoforms are present in humans; SCD1, which is expressed in most adult tissues, and SCD5, which is highly expressed in embryo tissues and adult brain [5], [6]. It has been shown that malignant transformation in lung cancer cells is positively correlated with SCD1 activity and expression [7]. Furthermore when several cancer cell lines were screened with a siRNA library against 3,700 genes to identify suitable targets for inducing cytotoxicity and cell death, SCD1 was one of the main targets identified [8]. In lung cancer cells, abrogation of SCD1 gene expression leads to impaired de novo lipid synthesis, a reduced rate of cell proliferation, a loss of anchorage-independent growth and higher rates of ceramide-independent apoptosis [9]. These findings strongly implicate SCD1 in the regulation of proliferation, invasiveness and survival of cancer cells.

SCD1 also plays a key role in tumor formation and growth. In mice, the background level of SCD1 expression correlates with predisposition to liver carcinogenesis; rodents with higher levels of SCD1 are more susceptible to induction of cancer [10]. Furthermore, using athymic “nude” mice, we demonstrated for the first time that lung cancer cells with reduced levels of SCD1 exhibit a severely impaired capacity for tumor formation and progression of tumor growth, suggesting that SCD1 is a critical factor in tumorigenesis [11].

We previously reported [9], [11], [12] that SCD1, by converting SFA into MUFA, regulates cancer cell lipogenesis by: i) maintaining ACC in its activate state though the conversion of saturated acylCoAs which are allosteric inhibitors of ACC into MUFA; ii) promoting the dephosphorylation and inactivation of AMPK, the main cancer cell fuel sensor that targets ACC for phosphorylation/inactivation; and iii) inducing the activation of the Akt pathway, which activates the expression of key lipogenic enzymes [3]. While these results clearly support SCD1 as a central regulator of lipogenesis in cancer cells, they do not fully explain how SCD1 and lipogenesis interact in regulating cancer cell mitogenesis and transformation.

The goal of the present study was to investigate the role of SCD1 in modulating cell cycle progression. Using a novel small molecule inhibitor of SCD activity, we determined that acute pharmacological inhibition of SCD1 in lung cells blocks the passage of cycling cells from G_1_ to S-phase and induces the entrance of cells into programmed cell death. Moreover, in cells incubated with a small molecule inhibitor of ACC, a rate-limiting lipogenic enzyme that is modulated by SCD1 activity levels, similar alterations in cell proliferation were observed. Consistent with fatty acid synthesis being the common target pathway, there was no synergistic cytostatic effect when cells were incubated with both an ACC and SCD inhibitor.

## Materials and Methods

### Materials

AG01518 normal human skin fibroblasts were obtained from Coriell (Camden, NJ). H460 human lung adenocarcinoma cells were from ATCC (Manassas, VA). Cell culture media and other culture reagents were from Invitrogen Life Technologies (Carlsbad, CA). [6-^3^H]thymidine was from American Radiolabeled Chemicals, Inc. (St. Louis, MO). Ultrafiltered fetal bovine serum (FBS), fatty acid-free bovine serum albumin (BSA), mouse anti-β-actin monoclonal antibody, anti-mouse IgG peroxidase conjugate, phosphatase and protease inhibitor cocktail were purchased from Sigma (St. Louis, MO). Cyclin D1 and CDK6 antibodies were from Cell Signaling Technology (Danvers, MA). Cell culture supplies, silica gel 60 chromatography plates, and analytical-grade solvents were from Fisher Scientific, (Morris Plains, NJ). CP-640186 was kindly donated by Donnie Owens, Pfizer.

### Cell culture

Cells were grown in DMEM supplemented with 10% FBS, penicillin (100 U/ml), streptomycin (10 µg/ml), 1% non essential amino acids and 1% MEM vitamin solution (growing medium), at 37°C, 5% CO_2_, and 100% humidity.

### Cell proliferation assay

Cell proliferation rate was determined in normal human fibroblasts and H460 cancer cells by Crystal violet assay [12]. Briefly, cells were fixed with methanol, stained with 0.1% crystal violet in distilled water and rinsed three times with water. The dye in the stained cells was solubilized in 10% methanol, 5% acetic acid solution and quantified by spectrophotometry at 580 nm. The value of a blank well was subtracted in each case. Results were expressed as percentage change in cell proliferation with respect to OD values of vehicle-treated control (100%). In these cells, SCD1 activity was abrogated by incubation with the small molecule SCD inhibitor CVT-11127 [13]. At the concentrations used in experiments, CVT-11127 inhibited SCD1 activity more than 95% [12]. Cells were also treated with 20 µM CP-640186, a potent inhibitor of ACC activity [14]. Cells were incubated with the inhibitors for 48 h or more in order to allow for at least one population doubling. For some experiments, exogenous fatty acids complexed with BSA (ratio 2∶1) were added to the incubation media.

### Determination of cell cycle distribution

In order to determine the distribution of cell populations in different phases of cell cycle, H460 cells were treated with either 1 µM CVT-11127, 20 µM CP-640186, or DMSO vehicle for 48 h. Groups of cells were incubated in parallel with 100 µM fatty acids complexed with BSA. At the end of incubations, cells were collected and treated with 50 µl RNase I (1 mg/ml) and stained with 5 ul propidium iodide (1 mg/ml). The percentage of cells in cell cycle phases was analyzed by fluorocytometry.

### Determination of apoptosis by DNA fragmentation assay

The determination of DNA fragmentation rate was performed as described by Scaglia & Igal [11]. Briefly, DNA in preconfluent cells was labeled with 0.4 µCi [^3^H]thymidine in regular growing medium for 24 h. Medium was then removed, the cell monolayers were washed twice with PBS at 37°C and cells were allowed to grow for 24 h in presence of 1 µM CVT-11127 or vehicle. Groups of cells were supplemented with oleic acid. After treatment, the chase medium containing detached apoptotic cells was collected and the [^3^H]radioactivity was determined in a scintillation counter. Cell monolayers were lysed in PBS with 1% Triton-X100 and 0.2 µM EDTA and sedimented by centrifugation. Radioactivity was quantified in the supernatant containing fragmented [^3^H]DNA, and in the pellet containing intact cellular DNA. The pellet was washed and resuspended in 1% Triton-X100 and 0.2 µM EDTA. The percentage of fragmented [^3^H]DNA was estimated according to the following calculation: (chase medium DPM + supernatant DPM)/total DPM.

### Cell lipid extraction and analysis

Cell lipids were extracted following the procedure of Bligh & Dyer [15]. Total phospholipids and individual neutral lipids were separated by thin-layer chromatography (TLC) as described in Scaglia and Igal [7]. Lipid spots on the TLC plate were stained with iodine vapors, photographed and their relative content was quantified by optical densitometry in Bio-Rad Chemidoc digital image system using QuantityOne software.

## Results

### SCD1 controls the passage of H460 cells through the G_1_/S boundary of the cell cycle

**Figure 1:**
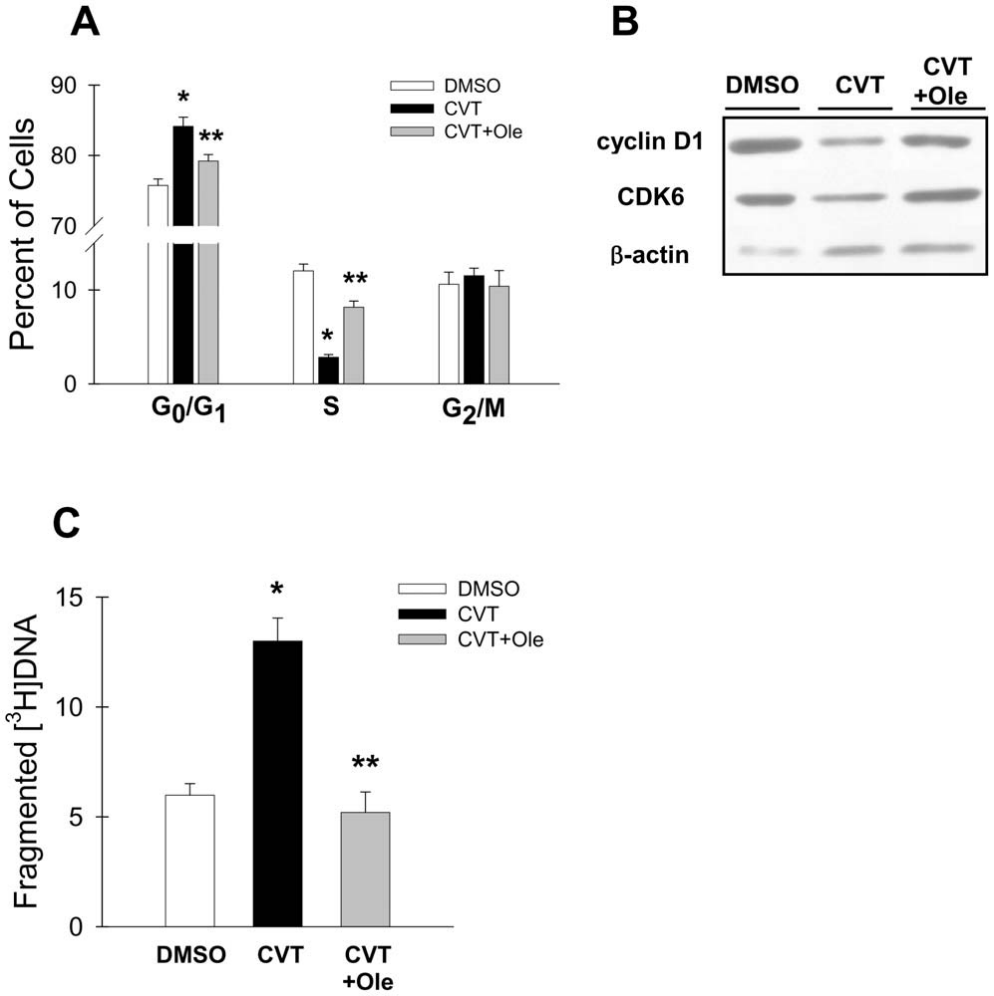
The inhibition of SCD1 activity blocks cell cycle progression and induces programmed cell death in cancer cells. In H460 lung cancer cells treated with 1µM CVT-11127 (CVT) or DMSO for 48h, in presence or absence of 100µM oleic (Ole), the distribution of cells in cell cycle phases was determined by fluorocytometry (**A**), levels of cyclin D1, CDK6 and β-actin were assessed by Western blot (**B**), and the rate of apoptosis was determined by the levels of fragmented [^3^H]thymidine-labeled DNA (**C**). *, p<0.05 or less vs. vehicle-treated cells; **, p<0.05 or less vs CVT-treated cells, by Student’s t test.

Previous studies from our laboratory established that acute and chronic depletion of SCD1 in cancer cells resulted in impaired cell proliferation [9], [11], [12]. To better understand the mechanism by which SCD1 inhibition impairs cell growth, H460 lung adenocarcinoma cells were incubated with 1 µM CVT-11127, a novel small molecule inhibitor of SCD1, in serum-containing media for 48 h and cell cycle progression was analyzed by flow cytometry (Fig. 1A). It was observed that the population of cells in S-phase was decreased by ∼75% with CVT-11127 treatment when compared to vehicle-treated controls, indicating that SCD1 inhibition specifically targets the progression of the cell cycle at the level of the synthetic phase. A concomitant increase in the population of SCD1-deficient cells in G_1_ phase was also detected, while there were no changes in percentage of cells in G_2_/M phase. However, in cells incubated with the SCD inhibitor in serum-deficient media, a ∼50% decrease in cells in G_2_/M-phase was also observed (data not shown), suggesting that identified components of serum, possibly MUFA-containing lipids, were able to sustain the passage of SCD1-deficient cells through mitosis. Overall, these results indicate that cycling cells with a blockade in SCD1 activity were not able to progress through the early stages of the cell cycle. Exogenous oleic acid markedly reversed the cell cycle changes produced by SCD1 inhibition demonstrating the critical importance of MUFA to cell cycle progression.

The levels of mammalian cyclins and cyclin-dependent kinases (CDKs) notably fluctuate during cell cycle progression. Cyclins D and CDKs are key determinants in the passage of the cell cycle through G_1_ phase, past the restriction point and into S-phase. Having observed that the pharmacological ablation of SCD1 promoted an alteration in the progression G_1_→S, we determined the levels of cyclin D1 and CDK6 when compared to vehicle-treated controls. We observed that after treating cells for 48 h with the SCD1 inhibitor, cancer cells exhibited a marked decrease in the content of both cyclin D1 and CDK6 (Fig. 1B). Incubation of SCD1-depleted cells with oleic acid normalized the levels of both proteins confirming that MUFA are crucial molecules in the regulation of the cell cycle.

Since the capacity of cancer cells to evade programmed cell death also contributes to the rate of cancer cell proliferation, the effects of oleic acid on the restoration of cell proliferation in the presence of an SCD1 inhibitor could be due, in part, to suppression of apoptosis. Therefore, we determined the rate of DNA fragmentation, a typical marker of apoptosis, in cells treated with CVT-11127 or DMSO for 48 h in the presence or absence of 100 µM oleic acid. As shown in Fig. 1C, fragmentation of DNA was increased by 2.2-fold in SCD1-ablated cells compared to controls, indicating that SCD1 is a key survival factor in cancer cells. We also observed that exogenous oleic acid reduced the level of fragmented DNA in SCD1-deficient cells to control values, suggesting that this fatty acid or a fatty acid product of SCD1 is essential for preventing apoptosis in cancer cells.

### Cis-MUFA rescue cell proliferation impaired by SCD1 inhibition

**Figure 2:**
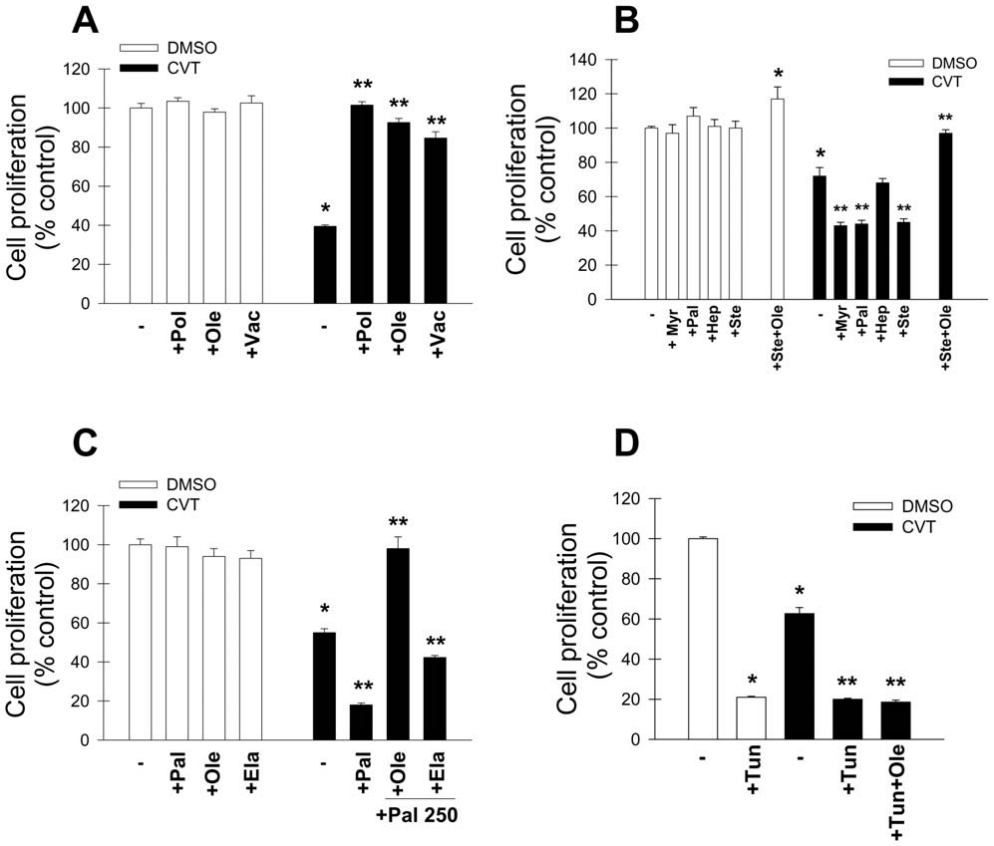
Impaired cell proliferation of cancer cells with a blockade in SCD1 activity is restored by exogenous cis- and trans-MUFA. **A**, H460 lung cancer cells were treated with 1 µM CVT-11127 (CVT) or DMSO for 48 h in presence or absence of 100 µM cis-MUFA palmitoleic acid (Pol), oleic acid (Ole) or cis-vaccenic acid; and cell proliferation was determined by Crystal violet staining. Proliferation was similarly assessed in H460 cells that were treated with 1 µM CVT-11127 (CVT) or DMSO for 48 h in presence or absence of 100 µM SFA myristic (Myr), palmitic (Pal), heptadecanoic (Hep), or stearic (Ste) acids (B), or 250 µM Pal, plus or minus 250 µM Ole or elaidic (Ela) (**C**). Groups of DMSO- and CVT-treated cells were incubated with 2 µg/mL tunicamycin in presence or absence of 100 µM oleic acid for 48 h and cell growth was determined (**D**). *, p<0.05 or less, vs DMSO; **, p<0.05 or less vs fatty acid-treated control, by Student's t test.

We have previously shown that the anti-proliferative effects of CVT-11127 (1 µM) could be reversed by oleic acid [12]. However, the effect of other SCD product fatty acids has not been investigated. In H460 lung cancer cells, we found that palmitoleic (16:1 n-7) and cis-vaccenic acids (18:1n-7), like oleic acid completely reversed the anti-proliferative effect of CVT-11127 (Fig. 2A). However, when H460 cells were incubated with or without 1 uM CVT or DMSO for 48 h in presence of either 100 µM myristic (14:0), palmitic (16:0), heptadecanoic (17:0), or stearic acids (18:0) (Fig. 2B), we observed that all the SFA, with the exception of heptadecanoic acid, a non-natural fatty acid, augmented the anti-proliferative effect of the SCD inhibitor, whereas the SFA had no anti-proliferative effect in cells treated with vehicle. In addition, the anti-proliferative effect of both SCD1 inhibition and stearic acid could be overcome by co-incubation with oleic acid. Taken together these observations clearly show that both endogenous and exogenous SFA are cytotoxic in the absence of either SCD to produce MUFA or exogenously added MUFA.

As shown in Fig. 2C, when CVT-treated cells or their controls were incubated with 250 µM palmitic acid cell proliferation decreased even further than in cells with a blockade in SCD1 and incubated with 100 µM palmitic acid. However, the cytotoxic effect of very high palmitic acid was fully prevented with oleic acid but only partially with 250 µM of the trans-MUFA elaidic acid, suggesting that only cis-MUFA are functionally appropriate to counteract the cytotoxic effect of high levels of SFA.

To determine whether MUFA provides broad protection against cytotoxicity, we determined the ability of oleic acid to overcome the anti-growth effect of tunicamycin, a compound that promotes cell stress and apoptosis by inhibiting protein glycosylation. Tunicamycin decreased cell proliferation by 80% in both CVT and vehicle-treated cancer cells, whereas addition of oleic acid was unable to restore the proliferation of SCD1-inhibited cells (Fig. 2D). This observation emphasizes that the protective action of SCD1 against SFA-mediated cytotoxicity in cancer cells results from its specific activity in the fatty acid biosynthetic pathway and not general cytoprotective activity.

### Blockade of SCD1 activity with CVT-11127 impairs the proliferation of H460 cancer cells but not normal human fibroblasts

**Figure 3:**
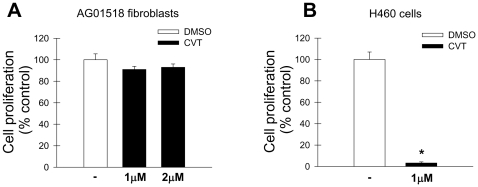
Inhibition of SCD activity with CVT-11127 impairs the proliferation of H460 cancer cells but not normal human fibroblasts. Cell growth was determined in AG01518 normal human fibroblasts (**A**) in presence of 1 and 2 µM CVT-11127 in 10%FBS DMEM or vehicle for 96 h. H460 cells (**B**) were treated with 1 µM CVT-11127 or vehicle for 96 h. Cell proliferation rate was assessed by Crystal violet staining. *, p<0.05 or less, vs DMSO; by Student's t test.

In earlier studies we reported that the proliferation of normal human skin fibroblasts was not impaired by SCD inhibition [12]. However, it is possible these cells are resistant to the effects of SCD inhibition and need to be incubated for a longer period of time with the inhibitor or with a higher concentration of inhibitor. To look at the effect of incubation time and inhibitor concentration, normal human fibroblasts were incubated for 96 h, time enough to ensure at least one population doubling, with 1 and 2 µM CVT-11127 in medium containing 10% FBS. As displayed in Fig. 3A, incubation with the SCD inhibitor did not effect the proliferation of these cells. However, similar conditions completely blocked (98%) the growth of H460 cells (Fig. 3B). Both normal and cancer cells were treated with the SCD inhibitor in serum-deficient media and the effect of the SCD inhibitor on cell growth was independent of the presence of serum (data not shown). The fact that fibroblasts were completely unresponsive to the cytostatic effect of the SCD inhibitor suggests that non-cancer cells have a smaller requirement for endogenously produced MUFA than cancer cells.

### Active de novo lipid synthesis is required for fatty acid-mediated reversal of the anti-proliferative effect of SCD1 inhibition

**Figure 4:**
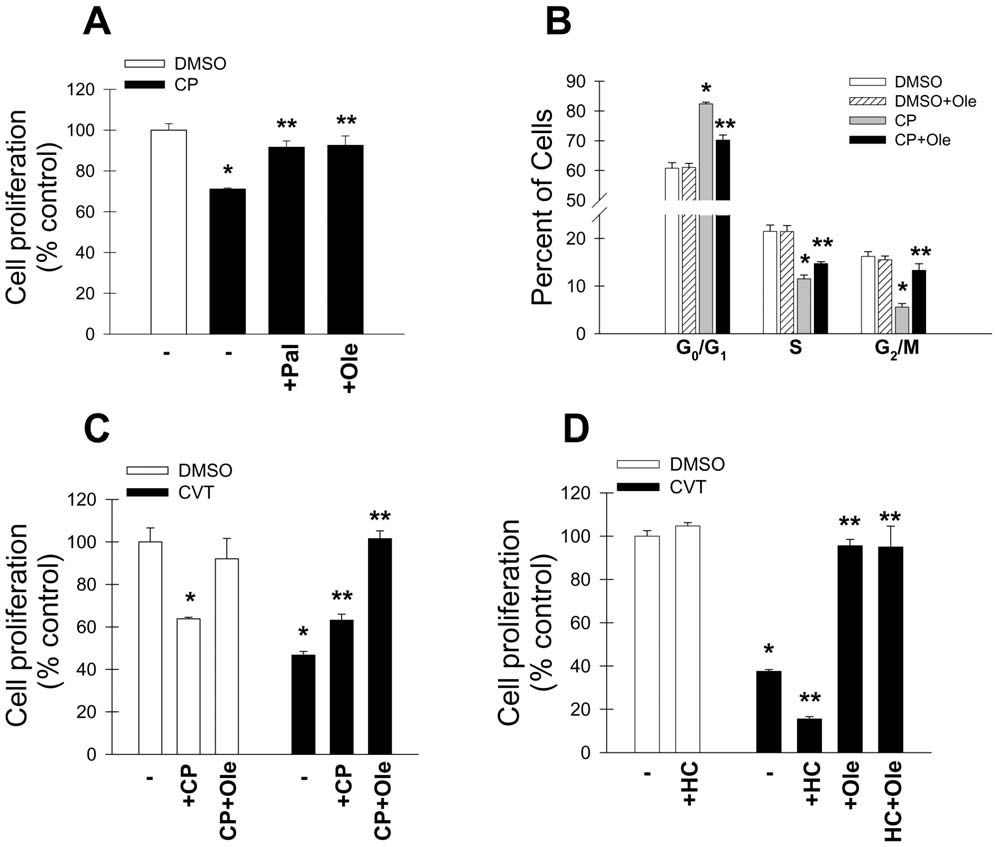
Oleic acid is unable to fully reverse the low proliferation rate of SCD1-deficient when de novo lipid synthesis is blocked. H460 lung cancer cells were treated with 1 µM CVT-11127 (CVT), 10 µM CP-640186 or both in presence or absence of 100 µM palmitic acid (Pal) or oleic acid (Ole) (**A**), and cell proliferation was assessed after 48 h. Distribution of cells in cell cycle phases was determined by fluorocytometry in cells incubated with CP-640186 plus or minus 100 µM oleic acid and (**B**). Cancer cell proliferation was also determined upon treatment with 1 µM CVT-11127 (CVT), 20 µM CP-640186, or both in presence or absence of 100 µM palmitic acid (Pal) or oleic acid (Ole) (**C**), and in cells treated for 48 h with 1 µM CVT-11127, 25-hydroxycholesterol or both, plus or minus 100 µM oleic acid (**D**). In all experiments, control cells received equivalent volumes of DMSO vehicle. *, p<0.05 or less compared to DMSO; **, p<0.05 or less compared to CVT-treated control, by Student's t test.

We have reported that inhibition of SCD1 reduces lipogenesis, at least partly by inactivating the rate-limiting lipogenic enzyme ACC-α and that reduced lipogenesis may contribute to the low proliferation by SCD1-ablated cells [12]. To investigate the relationship between SCD1, ACC and lipogenesis in cancer cell proliferation, we treated H460 cells with CP-640186, a specific inhibitor of ACC, at a concentration of 20 µM which inhibits more than 95% of enzyme activity [14]. As shown in Fig. 4A, incubation of cells with the ACC inhibitor for 48 h led to a ∼30% decrease in cell number compared to vehicle-treated controls. This is in agreement with previous reported studies [16], [17]. The cytostatic effect of ACC blockade was reversible by both 100 µM palmitic acid and 100 µM oleic acid (Fig. 4A) suggesting that ACC activity regulates cell proliferation by supplying fatty acids for lipid biosynthesis. The growth inhibitory effect of ACC inhibition was caused by a block in cell cycle progression since there was a significant reduction in the population of cells in S- and G_2_/M phases and a concomitant increase in cells in G_0_/G_1_ with respect to controls (Fig. 4B). Exogenous oleic acid had a marked effect on restoring the cell cycle profile of ACC-depleted cells, similar to the effect on SCD1 inhibited cells reinforcing the concept that the ultimate metabolic purpose of the concerted activation of ACC, FAS and SCD1 observed in cancer cells is the production of MUFA.

Interestingly, co-treatment with CVT-11127 and CP-640186 did not potentiate the growth inhibitory effect of each of these compounds, suggesting that inhibition of either ACC or SCD1 affects a common product of the pathway. Since SCD1 is later in the fatty acid biosynthetic pathway, MUFA are the fatty acid critical for cell proliferation. Indeed, in cells incubated with both ACC and SCD1 inhibitors, oleic acid was able to fully restore the low cell proliferation rate of H460 cells to control values. These data strongly support the conclusion that MUFA are the functional end product(s) of fatty acid biosynthesis that are required for cancer cell growth.

Lipid biosynthesis in mammalian cells is controlled by the transcriptional factors SREBP-1a and SREBP-1c, which are central regulators of fatty acid and phospholipid synthesis in mammalian cells [18], [19]. Induction of lipogenesis in human non-transformed and cancer cells requires SREBP-1 activation [20]–[22], therefore we determined the effect of down-regulation of lipogenesis by SREBP-1 inactivation on cell-proliferation. H460 cells were incubated with the SCD1 inhibitor for 48 h in presence or absence of 25-hydroxycholesterol, a potent inhibitor of SREBP-1 activation and cleavage by SCAP [19]. Incubation with 25-hydroxycholesterol alone did not affect cancer cell proliferation, however co-treatment of cells with the hydroxysterol and CVT-11127 promoted a more profound growth inhibitory effect than with the SCD1 inhibitor alone, an effect likely due to a potentiation of the anti-lipogenic activity of both inhibitors (Fig. 4D). Consistent with our previous observations, 100 µM oleic acid was sufficient to overcome the cytostatic effects of both SCD1 inhibition and combined SCD1 inhibition and down regulation of lipid synthesis, further confirming the importance of both lipid synthesis and fatty acid desaturation by SCD1 in providing the substrates for cancer cell growth.

**Figure 5:**
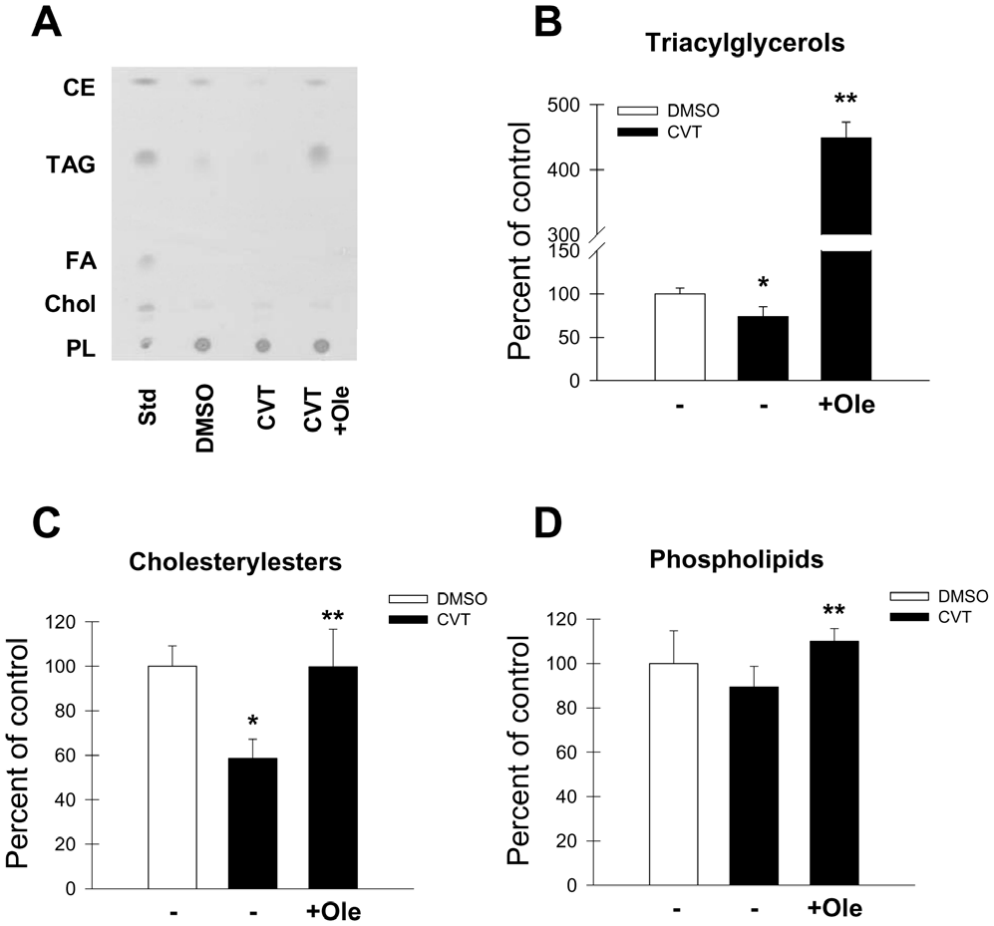
Effect of exogenous fatty acids on the levels of lipids in SCD1-deficient cells. H460 lung cancer cells were treated with 1 µM CVT-11127 (CVT) for 48 h in presence or absence of 100 µM oleic acid (Ole). Total cell lipids were extracted, separated by TLC and stained with iodine vapors (**A**). Relative levels of, triacylglycerols (**B**), cholesterolesters (**C**), and phospholipids (**D**), were determined by densitometric scanning. *, p<0.05 or less, vs DMSO; **, p<0.05 or less vs CVT-treated control, by Student's t test.

Finally, additional proof that active lipid formation is a key step in the regulation of cell proliferation by SCD1 was obtained by examining the relative content of major lipids in cells undergoing inhibition of SCD1 and treated with exogenous fatty acids. As displayed in Fig. 5A, blockade of SCD1 with CVT-11127 led to a significant reduction of the main neutral lipids, CE and TAG while total phospholipids were slightly reduced. Incubation of SCD1-depleted cells with 100 µM oleic acid dramatically increased the levels of TAG by 4.4-fold over control values (Fig. 5B). Addition of oleic acid fully restored the decreased content of CE in SCD1-deficient cells (Fig. 5C), whereas it returned phospholipid levels (Fig. 5D) to control values. Altogether, these observations reinforce the notion that SCD1 determines the rate of cell cycle progression and programmed cell death, and ultimately, the proliferation of cancer cells by sustaining active lipid synthesis and cis-MUFA production.

## Discussion

In previous studies, we reported that genetic and pharmacological ablation of SCD1 severely impairs the ability of cancer cells to proliferate [12]. In the present work, we provide new evidence that SCD1 controls the rate of cancer cell mitogenesis by modulating cell cycle progression. Our observation that cancer cells treated with a pharmacological inhibitor of SCD1 are arrested in the G_1_ phase and that this effect is restored by oleic acid suggests that MUFA synthesis is required in early phases of the cell cycle. Furthermore, inhibition of ACC and FAS, two key enzymes of the synthesis of fatty acids, elicited a similar blockade in the progression of the cell cycle through the G_1_/S boundary [16], [23], [24]. These observations suggest that a concerted activation of SFA synthesis and subsequent conversion of SFA into MUFA is required to provide the phospholipid biosynthetic machinery with MUFA substrates for new membrane synthesis before or during the synthetic phase of the cell cycle. In fact, elegant studies by Jackowski [25], [26] revealed that the accumulation of new phospholipids for dividing cells is the product of elevated synthesis and turnover that occurs during G_1_ and early S phases.

Our data also suggest that SCD1 may control cell cycle progression by altering the levels of cyclin D1 and CDK6, two proteins whose expression and interaction are critical for the passage of cycling cells through G_1_/S transition [27]. SCD1 activity may indirectly regulate the content of these cell cycle proteins by modulating GSK3β, a downstream target of the Akt pathway that increases the degradation of cyclin D1 [28]. We have previously reported that inhibition of SCD1 expression inactivates Akt and increases the dephosphorylation and activation of GSK3β. Changes in the biochemical composition and hence the biophysical properties of cellular membrane domains can activate signal transduction and transcription pathways that modulate mitogenesis [3]. Control of SFA and MUFA balance by SCD1 appears to be a critical modulator of growth signals in human cells [11], [12], however the mechanisms through which this enzyme coordinates modifications in membrane lipid composition, membrane-derived signals and their downstream effects are still not fully understood.

While cancer cells multiply through persistent cell division, the number of proliferating cells is also affected by the rate of cell death. Besides favoring a greater rate of cell mitogenesis, we provide evidence that SCD1 is an important survival factor for cancer cells by helping the cell avoid programmed cell death through production of cis-MUFA. SCD1 activity may prevent cancer cell apoptosis by at least two mechanisms: protection from SFA-mediated toxicity or lipoapoptosis [11], [29], and the stimulation of cis-MUFA biosynthesis for cell proliferation. High constitutive fatty acid synthesis is typically found in cancer cells [3], hence a tonically active SCD1 may prevent the potentially deleterious effects of endogenous SFA accumulation. Since elaidic acid, a trans-MUFA, only weakly prevented the cytotoxic effect of both SCD1-deficiency and SFA while cis-MUFA fully restored proliferation, there appears to be some fatty acid species specificity for the protective effect. This possibly occurs by MUFA displacing SFA from key cytotoxic metabolic reactions.

Active lipogenesis, particularly membrane lipid synthesis, is a critical requirement for the continuous proliferation of cancer cells and a mechanism for avoiding entry into the program of apoptosis [30]. We have previously established that SCD1 controls the overall rate of lipid synthesis in lung cancer cells [9], [11], [12]. Results from the present work reinforce the concept that SCD1 may control cell proliferation by affecting the fatty acid biosynthetic rate [12]. We observed a significant decrease in the proliferation of H460 cells in the presence of an ACC inhibitor and the anti-proliferative effect which can be attributed to defective fatty acid synthesis could be rescued by both oleic acid (MUFA) and palmitic acid (SFA). These findings agree with previous reports demonstrating that cell growth arrest in ACC depleted cells could be reversed by exogenous palmitic acid [16], [17]. Since both ACC and SCD1 inhibition promoted similar alterations to cell cycle progression that were reversible by exogenous oleic acid, it is reasonable to conclude that MUFA are the ultimate functional product in cancer cells necessary for rapid cell proliferation and resistance to SFA cytotoxicity.

Our data show that exogenously added oleic acid restored neutral lipid levels in SCD deficient cells. The pro-lipogenic effect of MUFA may be due to their capacity to act as substrates for acylation as it has been established that MUFA are preferred over SFA as substrates for triacylglycerol and cardiolipin synthesis [31], [32]. In cancer cells, the high MUFA production promoted by SCD1 activity ensures the overactive lipid biosynthetic machinery is supplied with preferential substrates. The presence of highly unsaturated lipids in cancer cells may have critical implications for their biological phenotype. The constitutive activation of SCD1 in cancer cells enriches the major phospholipids of cell membranes with MUFA thereby producing a more fluid lipid membrane environment [7], a condition that is thought to induce growth factor-activated proliferation [33], [34], cancer growth and invasiveness [35]–[37].

In conclusion, this is the first published study demonstrating that SCD1 modulates the passage of cycling cells through the G_1_/S boundary and the entry in the apoptotic program. This work supports the view that SCD1 regulates mitogenesis by modulating the rate of fatty acid synthesis, by preventing the toxic accumulation of SFA, and by controlling the supply of MUFA substrates required for lipid biosynthesis and cancer cell proliferation.
